# Supplementary material for: All-at-once RNA folding with 3D motif prediction framed by evolutionary information
Source: Nat Methods. 2025 Oct 3;22(10):2094–106. doi: 10.1038/s41592-025-02833-w (PMC12510878; doi:10.1038/s41592-025-02833-w)
Supplement: Supplementary file 3 — Source code to run CaCoFold-R3D. Data and results. [file 41592_2025_2833_MOESM3_ESM.gz › supplemental_material/results/CaCoFold-R3D/CaCoFold-R3D_Rfam_vshuffle/RF01711_Lnt.R2R.sto.pdf]

RF01711_Lnt

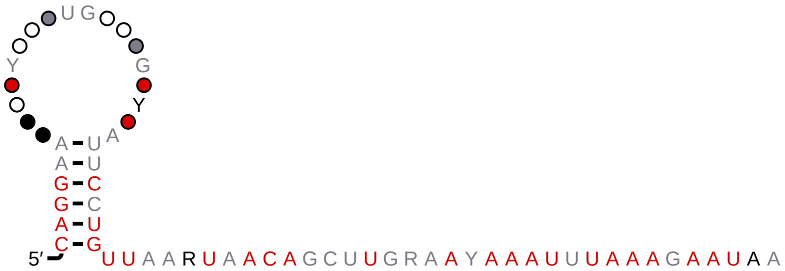

5′
U G
Y
G
Y
A
A U
A U
G C
G C
A U
C G
U U A A R U A A C A G C U U G R A A Y A A A U U U A A A G A A U A A
